# Supplementary material for: A genome-wide in vivo screen reveals fitness pathways required for streptococcal infective endocarditis
Source: PLoS Pathog. 2026 Aug 17;22(8):e1014156. doi: 10.1371/journal.ppat.1014156 (PMC13506083; doi:10.1371/journal.ppat.1014156)
Supplement: Supplementary file 1 [file ppat.1014156.s020.pdf]

1. Journal Requirements:

1) *Please ensure that the CRediT author contributions listed for every co-author are completed accurately and in full. At this stage, the following Authors/Authors require contributions: Liang Bao, Jennifer Bradley, Vysakh Anandan, Katarzyna Tyc, Zan Zhu, Josephina Anna Vossen, Valery francine Assi, Jasmine Benbei, Nicai Zollar, Todd Kitten, and Ping Xu. Please ensure that the full contributions of each author are acknowledged in the "Add/Edit/Remove Authors" section of our submission form. The list of CRediT author contributions may be found here: https://journals.plos.org/plospathogens/s/authorship#loc-author-contributions*

>> We have completed and updated the CRediT author contribution statements for all co-authors in the "Add/Edit/Remove Authors" section of the submission system

2) We ask that a manuscript source file is provided at Revision. Please upload your manuscript file as a .doc, .docx, .rtf or .tex. If you are providing a .tex file, please upload it under the item type u2018LaTeX Source Fileu2019 and leave your .pdf version as the item type u2018Manuscriptu2019.

>> We have uploaded the manuscript source file in .docx format as requested.

3) Please provide an Author Summary. This should appear in your manuscript between the Abstract (if applicable) and the Introduction, and should be 150-200 words long. The aim should be to make your findings accessible to a wide audience that includes both scientists and non-scientists. Sample summaries can be found on our website under Submission Guidelines: https://journals.plos.org/plospathogens/s/submission-guidelines#loc-parts-of-a-submission

>> We have added an Author Summary to the revised manuscript.

4) Please upload all main figures as separate Figure files in .tif or .eps format. For more information about how to convert and format your figure files please see our guidelines: https://journals.plos.org/plospathogens/s/figures

>> We have uploaded all main figures as separate TIFF files.

5) We have noticed that you have uploaded Supporting Information files, but you have not included a list of legends. Please add a full list of legends for your Supporting Information files after the references list.

>> We have added a complete list of legends for all supplementary figures and supporting information files following the References section of the revised manuscript.

6) Some material included in your submission may be copyrighted. According to PLOS's copyright policy, authors who use figures or other material (e.g., graphics, clipart, maps) from another author or copyright holder must demonstrate or obtain permission to publish this material under the Creative Commons Attribution 4.0 International (CC BY 4.0) License used by PLOS journals. Please closely review the details of PLOS's copyright requirements here…- Figure 1: Please confirm whether you drew the images / clip-art within the figure panels by hand. If you did not draw the images, please provide (a) a link to the source of the images or icons and their license / terms of use; or (b) written permission from the copyright holder to publish the images or icons

under our CC BY 4.0 license. Alternatively, you may replace the images with open source alternatives.

>> Figure 1 consists of original graphics created by the authors using BioRender.com. We have clarified with a statement in legend: "Figure 1 was created with BioRender.com."

7) Please amend your detailed Financial Disclosure statement. This is published with the article. It must therefore be completed in full sentences and contain the exact wording you wish to be published.

>> We have added a separate "Financial Disclosure" section to the manuscript as follows: "Financial Disclosure statement. This work was funded by grants from the National Institutes of Health R01DE030121 (P.X. and T.K.), R03DE034511 (L.B.) and the CCTR Endowment Fund (P.X.). VCU Massey Comprehensive Cancer Center Bioinformatics Shared Resource is supported, in part, with funding from NIH-NCI Cancer Center Support Grant P30 CA016059.The funders had no role in study design, data collection and analysis, decision to publish, or preparation of the manuscript."

2. Reviewers' Comments: (Reviewer's Responses to Questions)

Part I – Summary (Please use this section to discuss strengths/weaknesses of study, novelty/significance, general execution and scholarship.)

Reviewer #1: This manuscript describes the search for a nonbiased list of genes that contribute to infective endocarditis (IE) caused by oral streptococci. This is a medically complex disease that is difficult to treat. The discovery of novel targets for therapies would be welcome. Previous work has focused on this problem is clearly recognized by the authors. They then openly discuss the many possible bottlenecks and limitations of the many studies using this validated animal model and develop a strategy to more broadly ask the question of how the endocardial lesions are colonized and infection progresses. The approach is intriguing and the results indicate success in developing a much wider genetic understanding of the fitness factors that promote disease than previously attained. The study initially uses strep sanguinis and is then repeated in the similar pathogen strep mutans. The genes are also compared across other pathogens that cause endocarditis but in more limited settings. The fact the there is considerable overlap lends support to their conclusions. They also look for compensatory mutations that arise during passage of knock out mutants. This is a clever approach to further support validation. This study is well thought out and the conclusions are robust, rigorously tested and, importantly, do not go beyond the data.

>> We thank the reviewer for the thoughtful evaluation of our work and for recognizing the technical rigor, experimental design, and careful interpretation of the data. We greatly appreciate the reviewer's recognition of the robustness of the study and the significance of the genome-wide approach for identifying IE-associated fitness determinants.

Reviewer #2: Bao et al present a genetic screen to identify genetic determinants of S. sanguinis infective endocarditis in an animal model. The strategy of repeat testing of mutants in different pools adds robustness to the study. An additional strength is the confirmation of these putative virulence factors in an S. mutans IE model. The attempt to isolate the mechanism of action for these putative virulence factors by comparing growth in rich media and in serum to the in vivo colonization defects was not as well supported, as many of the putative IE virulence factors had

reduced growth in rich media, suggesting that they may be generally attenuated, instead of specifically attenuated in the IE model.

>> We appreciate this important point. Our goal was to distinguish genes specifically contributing to IE-associated fitness from those generally affecting bacterial growth. To address this, we systematically evaluated growth phenotypes in rich BHI medium. Our results showed that many of the mutants had reduced growth in BHI (Figure 3C). This is most apparent in Fig. 3A vs. 3B. However, among the 137 IE fitness factors evaluated, 112 or 81.8% of the mutants were more deficient in the IE model than they were in BHI, even when their growth was significantly reduced in BHI.

We have now clarified this in our Results section "Of the 146 IE fitness determinant mutants identified *in vivo*, 137 were represented across the four pools and evaluated for growth (Figure 3B and 3C). Among these, 65 mutants exhibited reduced competitive fitness in BHI (abundance ratio <0.5) (Figure 3C), including two genes annotated as hypothetical proteins. Notably, 112 mutants were more severely attenuated in IE than in BHI. This subset included all six components of the shikimate pathway, as well as components for CoA and serine synthesis, the two core PTS components (EI and HPr), the SsaACB Mn^{2+} transporter, and at least two of the three ECF transporters. Conversely, 25 mutants—including most components related to *rml/rgp*—exhibited a greater growth reduction in BHI relative to the IE model (Figure 3C)."

Reviewer #2: The attempt to identify suppressor mutants through experimental evolution was a strength of the study, however the failure to test the experimentally evolved strains in the IE model was a weakness.

>> We thank the reviewer for this important comment. The primary objective of this study was to establish a genome-wide map of genes associated with IE fitness. Although we demonstrated restoration of in vitro growth through experimental evolution, we did not further evaluate the evolved suppressor strains in the rabbit IE model in the current study. We have added this point as a limitation in the revised Discussion and agree that determining whether suppressor evolution restores cardiac vegetation colonization represents an important future direction.

In the Discussion section, in the first paragraph, we added "Second, while experimental evolution successfully identified suppressor mutations that restored *in vitro* growth in key mutant strains, these evolved strains were not evaluated *in vivo*, leaving it an open question whether this phenotypic rescue translates to restored cardiac vegetation colonization." Also in the closing paragraph "Although experimental evolution identified suppressor mutations that restored *in vitro* growth, these strains were not evaluated in the rabbit IE model. Consequently, whether this phenotypic rescue translates to restored cardiac vegetation colonization remains unknown. Testing the *in vivo* fitness of these evolved strains represents an important future direction to determine if niche-specific restrictions can be bypassed via compensatory evolution."

Reviewer #2: Further, while the authors suggest the importance of the identified factors as being important for infective endocarditis, the experiments do not adequately differentiate between the ability for the bacteria to survive in the blood versus the ability for the bacteria to colonize the endocardium.

>> We thank the reviewer for raising this important point in two separate comments. In our rabbit IE model, bacteremia is highly transient following inoculation, and most bacteria are rapidly cleared from circulation. We examined bacterial counts in peripheral blood samples collected during infection and found that only minimal CFU levels could be recovered from the bloodstream.

We have added statements in the first paragraph of the Discussion "While we did not compare mutant frequencies in peripheral blood versus cardiac vegetations, our previous work in this rabbit model demonstrates that circulating blood CFU counts are typically at least 5 logs lower than those in vegetations (PMID: 22039480). This dramatic difference creates a severe biological bottleneck, rendering peripheral blood bacterial yields too low for meaningful pooled frequency comparisons; thus, while our findings focus on the definitive pathological site of IE, future single-strain studies are required to fully differentiate between endocardial colonization and standalone bloodstream survival."

Reviewer #2: Additionally, the authors discuss the conservation of these IE virulence factors in many IE pathogens as support for these being genetic determinants of IE pathogenesis, however, this argument is weakened by the failure to show that these genetic determinants are absent from bacterial pathogens that do not cause endocarditis.

>> We thank the reviewer for raising this critical point regarding off-target toxicity and broad distribution within commensal or non-endocarditis strains. To address this concern, we significantly expanded our comparative genomic analysis. We curated a control dataset of 73 low-IE-risk or commensal bacterial strains, including 60 strains from five non-endocarditis *Streptococcus* species (*S. pneumoniae*, *S. agalactiae*, *S. pyogenes*, *S. dysgalactiae*, and *S. anginosus*) as well as 13 non-streptococcal strains that commonly colonize the gastrointestinal tract or other mucosal surfaces (including *Haemophilus influenzae* and others); listed in Table S7. We compared these against 113 high-IE-risk strains spanning the pathogenic genera *Streptococcus*, *Enterococcus*, and *Staphylococcus*. Our statistical analysis revealed that these target genes are widely conserved across both groups, indicating they represent core metabolic or evolutionary frameworks rather than niche-specific infective endocarditis virulence factors. We have updated the Results section to include this statistical evaluation.

We have revised the Results section as follows: "To determine whether this widespread conservation reflects IE-specific adaptations or general bacterial essentiality, we expanded our comparative genomic analysis to include 73 low-IE-risk control strains (comprising multiple strains of non-endocarditis Streptococcus species (PMID:37760749; 38973607; 32580572;) and 13 non-streptococcal bacterial species (PMID: 38247592; 34603862; 20218045; 37064722; 33134201; 38488973; 26632711; 26632711; 18087053)). We observed no significant difference in the overall conservation of the IE fitness genes between the 113 IE-associated pathogens and the 73 low-IE-risk strains; while 105 of the 145 fitness genes were present in >50% of IE-associated strains, 98 of these genes met the same conservation threshold among the low-risk pathogens (Fisher's exact test, $p = 0.38$). Collectively, these data suggest that while these core functions are required for IE, their broad conservation reflects a shared Gram-positive evolutionary background rather than IE-specific adaptation. Thus, the conserved IE fitness architecture comprises general essential functions integrated with species-specific accessory systems."

Reviewer #3: This is a strong and technically ambitious manuscript presenting the first genome-wide in vivo fitness analysis of Streptococcus sanguinis IE using a vertebrate model. The study provides a substantial dataset that offers a coherent physiological insight into the pathways required for the pathogen to establish IE. While I appreciate the technical rigor and experimental care the authors presented in this story, I do have several minor comments I wish the authors would consider, and mostly regarding conceptual framing and resolving clarities.

>> We sincerely thank the reviewer for their positive assessment and encouraging comments regarding the technical rigor, experimental care, and the overall value of our genome-wide *in vivo* fitness dataset. We appreciate the constructive feedback on our conceptual framing and clarity.

We have addressed each of the minor comments thoroughly below and revised the manuscript accordingly to ensure maximum clarity.

Part II – Major Issues: Key Experiments Required for Acceptance

Please use this section to detail the key new experiments or modifications of existing experiments that should be absolutely required to validate study conclusions.

Generally, there should be no more than 3 such required experiments or major modifications for a "Major Revision" recommendation. If more than 3 experiments are necessary to validate the study conclusions, then you are encouraged to recommend "Reject".

Reviewer #1: None

>> We thank the reviewer for the assessment.

Reviewer #2: The experiments as designed cannot differentiate between survival and growth in the bloodstream versus ability to colonize the endocardium. If the authors could take peripheral blood from the infected animals at the time of tissue harvest and compare relative frequency of mutants in the blood versus in the vegetation, that would support that the identified virulence factors are specific to IE and not general sepsis virulence factors. Alternatively, to reduce animal numbers, growth of libraries in whole blood (rabbit, or even human to increase the relevance of the model to human disease) would be acceptable.
>> We thank the reviewer for raising this important point in two separate comments. In our rabbit IE model, bacteremia is highly transient following inoculation, and most bacteria are rapidly cleared from circulation. We examined bacterial counts in peripheral blood samples collected during infection and found that only minimal CFU levels could be recovered from the bloodstream.

We have added statements in the first paragraph of the Discussion "While we did not compare mutant frequencies in peripheral blood versus cardiac vegetations, our previous work in this rabbit model demonstrates that circulating blood CFU counts are typically at least 5 logs lower than those in vegetations (PMID: 22039480). This dramatic difference creates a severe biological bottleneck, rendering peripheral blood bacterial yields too low for meaningful pooled frequency comparisons; thus, while our findings focus on the definitive pathological site of IE, future single-strain studies are required to fully differentiate between endocardial colonization and standalone bloodstream survival."

As the putative IE virulence factors are involved in essential functions, it is not surprising that there is a high degree of conservation with other taxa. The authors assert that finding these putative virulence factors in many bacterial strains that cause IE supports the role of these gene products in IE. However, unless they authors could show a lack of conservation of these factors in bacteria that do not cause IE, this conservation is merely coincidental.
>> We thank the reviewer for raising this critical point regarding off-target toxicity and broad distribution within commensal or non-endocarditis strains. To address this concern, we significantly expanded our comparative genomic analysis. As mentioned above, we curated a control dataset of 73 low-IE-risk or commensal bacterial strains, including 60 strains from five non-endocarditis *Streptococcus* species (*S. pneumoniae*, *S. agalactiae*, *S. pyogenes*, *S. dysgalactiae*, and *S. anginosus*) as well as 13 non-streptococcal strains that commonly colonize the gastrointestinal tract or other mucosal surfaces (including *Haemophilus influenzae* and

others). We compared these against 113 high-IE-risk strains spanning the pathogenic genera *Streptococcus*, *Enterococcus*, and *Staphylococcus*. Our statistical analysis revealed that these target genes are widely conserved across both groups, indicating they represent core metabolic or evolutionary frameworks rather than niche-specific infective endocarditis virulence factors. We have updated the Results section to include this statistical evaluation.

We have added the following text to the Results section "To determine whether this widespread conservation reflects IE-specific adaptations or general bacterial essentiality, we expanded our comparative genomic analysis to include 73 low-IE-risk control strains (comprising multiple strains of non-endocarditis Streptococcus species (PMID:37760749; 38973607; 32580572;) and 13 non-streptococcal bacterial species (PMID: 38247592; 34603862; 20218045; 37064722; 33134201; 38488973; 26632711; 26632711; 18087053)). We observed no significant difference in the overall conservation of the IE fitness genes between the 113 IE-associated pathogens and the 73 low-IE-risk strains; while 105 of the 145 fitness genes were present in >50% of IE-associated strains, 98 of these genes met the same conservation threshold among the low-risk pathogens (Fisher's exact test, $p = 0.38$). Collectively, these data suggest that while these core functions are required for IE, their broad conservation reflects a shared Gram-positive evolutionary background rather than IE-specific adaptation. Thus, the conserved IE fitness architecture comprises general essential functions integrated with species-specific accessory systems."

Lines 231-252: the in vitro growth defects seen do not support the roles of these as IE virulence factors, instead they seem to cause a general attenuation in growth.
>> We appreciate this important point. Our goal was to distinguish genes specifically contributing to IE-associated fitness from those generally affecting bacterial growth. To address this, we systematically evaluated growth phenotypes in rich BHI medium. Our results showed that many of the mutants had reduced growth in BHI (Figure 3C). This is most apparent in Fig. 3A vs. 3B. However, among the 137 IE fitness factors evaluated, 112 or 81.8% of the mutants being more deficient in the IE model than they are in BHI, even when they are significantly reduced in BHI.

We have now clarified this in our Results section "Of the 146 IE fitness determinant mutants identified *in vivo*, 137 were represented across the four pools and evaluated for growth (Figure 3B and 3C). Among these, 65 mutants exhibited reduced competitive fitness in BHI (abundance ratio <0.5) (Figure 3C), including two genes annotated as hypothetical proteins. Notably, 112 mutants were more severely attenuated in IE than in BHI. This subset included all six components of the shikimate pathway, as well as components for CoA and serine synthesis, the two core PTS components (EI and HPr), the SsaACB Mn^{2+} transporter, and at least two of the three ECF transporters. Conversely, 25 mutants—including most components related to *rml/rgp*—exhibited a greater growth reduction in BHI relative to the IE model (Figure 3C)."

The details of generation of the S. mutans deletion strains are not present in the materials and methods section, and should be provided.

>> We thank the reviewer for identifying this omission. We have added detailed descriptions of *S. mutans* deletion mutant construction procedures to the Materials and Methods section.

The differences in in vitro growth attenuation between the S. sanguinis and the S. mutans shikimate mutants does not support the overall conclusions of the story. I suggest focusing on the coaC which had similar phenotypes in both species to better support the overall conclusions of this paper.
>> We thank the reviewer for this helpful suggestion. We agree that *coaC* represents one of the

strongest examples of conserved IE-associated fitness phenotypes between *S. sanguinis* and *S. mutans*. We have revised the Discussion sections to more clearly emphasize the conserved phenotype associated with *coaC* disruption in both species. "The fact that *coaC* disruption yields remarkably similar, attenuated fitness phenotypes in both *S. sanguinis* and *S. mutans* strongly reinforces our core conclusion: certain core metabolic modules are universally required for streptococcal survival and adaptation within the hostile endocardial niche. By focusing heavily on these shared, validated functional nodes, we can distinguish baseline species-specific baseline adaptations from the overarching, fundamental traits required to drive infective endocarditis."

While the experimental evolution for both the S. mutans shikimate mutants and the S. sanguinis CoA mutants demonstrate restoration of in vitro growth, to support the conclusion of these suppressor mutants being important in IE, the restoration of the ability to colonize cardiac vegetations is necessary. The evolved isolates should be tested in the IE model.

>> We thank the reviewer for this important suggestion. We agree that testing experimentally evolved suppressor strains in the rabbit IE model would provide additional mechanistic insight into the restoration of IE-associated fitness. We have acknowledged this limitation and included it as an important future direction in the revised Discussion.

"Although experimental evolution identified suppressor mutations that restored *in vitro* growth, these strains were not evaluated in the rabbit IE model. Consequently, whether this phenotypic rescue translates to restored cardiac vegetation colonization remains unknown. Testing the *in vivo* fitness of these evolved strains represents an important future direction to determine if niche-specific restrictions can be bypassed via compensatory evolution."

Reviewer #3: I don't think additional experiments are required for this work.

>> We thank the reviewer for the feedback.

Part III – Minor Issues: Editorial and Data Presentation Modifications
Please use this section for editorial suggestions as well as relatively minor modifications of existing data that would enhance clarity.

Reviewer #1: One question arises out of curiosity and could be more thoroughly discussed. The comparison of genomes of a variety of species known to cause endocarditis yields similarities. Does the reverse also hold true? Do species that rarely cause endocarditis not have the right "equipment"? or is this disease reliant on flexibility of changing metabolism as shown by the compensatory mutation results rather than a specific gene set?

>> >> We thank the reviewer for raising this critical point regarding off-target toxicity and broad distribution within commensal or non-endocarditis strains. To address this concern, we significantly expanded our comparative genomic analysis. We curated a control dataset of 73 low-IE-risk or commensal bacterial strains, including 60 strains from five non-endocarditis *Streptococcus* species (*S. pneumoniae*, *S. agalactiae*, *S. pyogenes*, *S. dysgalactiae*, and *S. anginosus*) as well as 13 non-streptococcal strains that commonly colonize the gastrointestinal tract or other mucosal surfaces (including *Haemophilus influenzae* and others). We compared these against 113 high-IE-risk strains spanning the pathogenic genera *Streptococcus*, *Enterococcus*, and *Staphylococcus*. Our statistical analysis revealed that these target genes are widely conserved across both groups, indicating they represent core metabolic or evolutionary

frameworks rather than niche-specific infective endocarditis virulence factors. We have updated the Results section to include this statistical evaluation.

We have revised the Results section "To determine whether this widespread conservation reflects IE-specific adaptations or general bacterial essentiality, we expanded our comparative genomic analysis to include 73 low-IE-risk control strains (comprising multiple strains of non-endocarditis Streptococcus species (PMID:37760749; 38973607; 32580572;) and 13 non-streptococcal bacterial species (PMID: 38247592; 34603862; 20218045; 37064722; 33134201; 38488973; 26632711; 26632711; 18087053)). We observed no significant difference in the overall conservation of the IE fitness genes between the 113 IE-associated pathogens and the 73 low-IE-risk strains; while 105 of the 145 fitness genes were present in >50% of IE-associated strains, 98 of these genes met the same conservation threshold among the low-risk pathogens (Fisher's exact test, $p = 0.38$). Collectively, these data suggest that while these core functions are required for IE, their broad conservation reflects a shared Gram-positive evolutionary background rather than IE-specific adaptation. Thus, the conserved IE fitness architecture comprises general essential functions integrated with species-specific accessory systems."

Would a mutant in transformation pathway so well known for these streps be a key part of the picture?

>>This is an interesting question. In a previous study (PMID: 22039480), we examined mutants deleted for the regulators required for expression of the "late" competence genes or both the early and late competence genes. Both mutants were essentially non-transformable; yet, neither mutant demonstrated IE fitness defects in the rabbit model. Moreover, neither of these mutants was identified as reduced in IE fitness in the current study. We have now added discussion of these findings to the revised Discussion section to address this point.

In Discussion: "Likewise, the transformation machinery itself appears dispensable for *in vivo* success. We previously demonstrated that mutants lacking key regulators of early or late competence genes were non-transformable yet retained wild-type fitness in a rabbit model of infective endocarditis (PMID: 22039480). Consistent with those findings, these transformation-deficient mutants showed no fitness attenuation in the current study, suggesting that competence pathways do not directly drive competitiveness in this context."

Reviewer #2: Line 32: As antibiotic discovery is not tested in this study, this statement could be removed from the abstract.

>>We thank the reviewer for this suggestion. We have removed statements related to antibiotic discovery from the Abstract to better align the text with the scope of the present study.

Line 70: Could the authors define what cardiac conditions elevate the risk of endocarditis? Also could the authors mention the elevated risk in injection drug users.

>>We thank the reviewer for this helpful suggestion. We have revised the Introduction to clarify major clinical risk factors associated with infective endocarditis, including age, prosthetic heart valves, congenital heart disease, and injection drug use, with appropriate supporting citations. "Clinical risk factors that significantly elevate the likelihood of developing infective endocarditis include advanced age, the presence of prosthetic heart valves, and underlying congenital heart diseases, as well as behavioral risk profiles such as injection drug use."

Lines 143 and 145: is this the wild type strain or the ssa_0169 mutant?

>> It is the SSA_0169 mutant. This mutant was tested in a previous study that showed no detectable fitness defect in vitro or in IE vegetations (PMID: 19423626).

Line 270: is SMU277 a homolog of ssa_0169? Or does it just have a similar phenotype?

>> SMU277 was not a homolog of ssa_0169. SMU277 was selected because prior Tn-seq analysis indicated that it did not display a detectable growth defect in rich medium (PMID: 29435491). We have clarified to the revised manuscript.

Line 180: Why were these mutants absent from the input library? Are they essential genes?
>> We thank the reviewer for this important question. The missing mutants were not essential genes. During preparation of the inoculum pools, these mutants were cultured individually; however, 37 mutants consistently showed poor recovery or unstable growth from the archived mutant library stocks and therefore could not be reliably incorporated into the final input pools. We have clarified this point in the added discussion of this technical limitation in the revised manuscript in the first paragraph of the Discussion.

"…our baseline screen was bounded by technical library constraints, as 37 non-essential mutants could not be evaluated due to poor recovery or unstable growth from archived stocks during inoculum preparation."

Line 224: is 39C typical of human infections or only the rabbit model?

>> We thank the reviewer for this question. Fever is a common clinical feature of human infective endocarditis, with temperatures exceeding 38°C reported in approximately 98% of patients (PMID: 19273776). In our rabbit IE model, infected animals commonly reached temperatures near 39°C, which is consistent with systemic febrile responses observed during human infection. We have clarified this point in the revised manuscript "This temperature models the systemic febrile response common to human infective endocarditis, where fevers exceeding 38°C occur in nearly 98% of cases (PMID: 19273776).".

Line 248: a citation of the availability of serine in cardiac vegetations is necessary.

>> We thank the reviewer for this suggestion. We have added citations regarding serine availability in rich medium (PMID: 11418553) and the metabolic environment of cardiac vegetations (PMID: 18656529). The revised text now states:
"...consistent with sufficient serine availability in rich medium (PMID: 11418553) but potentially limited serine availability within cardiac vegetations (PMID: 18656529)."

Paragraph beginning line 406: Leveraging these putative virulence factors as a target for design of rationale therapeutics opens the concern for toxicity to the microbiome, which is not addressed. The authors state high conservation of these putative genes in Streptococci and Enterococci, but these taxa are also common colonizers of the GI tract, and the authors should address if these candidate genes are present in GI colonizing strains.

>> We thank the reviewer for raising this critical point regarding off-target toxicity and broad distribution within commensal or non-endocarditis strains. To address this concern, we

significantly expanded our comparative genomic analysis. We curated a control dataset of 73 low-IE-risk or commensal bacterial strains, including 60 strains from five non-endocarditis *Streptococcus* species (*S. pneumoniae*, *S. agalactiae*, *S. pyogenes*, *S. dysgalactiae*, and *S. anginosus*) as well as 13 non-streptococcal strains that commonly colonize the gastrointestinal tract or other mucosal surfaces (including *Haemophilus influenzae* and others). We compared these against 113 high-IE-risk strains spanning the pathogenic genera *Streptococcus*, *Enterococcus*, and *Staphylococcus*. Our statistical analysis revealed that these target genes are widely conserved across both groups, indicating they represent core metabolic or evolutionary frameworks rather than niche-specific infective endocarditis virulence factors. We have updated the Results section to include this statistical evaluation.

We have revised the Results section "To determine whether this widespread conservation reflects IE-specific adaptations or general bacterial essentiality, we expanded our comparative genomic analysis to include 73 low-IE-risk control strains (comprising multiple strains of non-endocarditis Streptococcus species (PMID:37760749; 38973607; 32580572;) and 13 non-streptococcal bacterial species (PMID: 38247592; 34603862; 20218045; 37064722; 33134201; 38488973; 26632711; 26632711; 18087053)). We observed no significant difference in the overall conservation of the IE fitness genes between the 113 IE-associated pathogens and the 73 low-IE-risk strains; while 105 of the 145 fitness genes were present in >50% of IE-associated strains, 98 of these genes met the same conservation threshold among the low-risk pathogens (Fisher's exact test, $p = 0.38$). Collectively, these data suggest that while these core functions are required for IE, their broad conservation reflects a shared Gram-positive evolutionary background rather than IE-specific adaptation. Thus, the conserved IE fitness architecture comprises general essential functions integrated with species-specific accessory systems."

Lines 462 and 482: could the authors report centrifugation conditions as xg rather than RPM which can vary from unit to unit.

>> We thank the reviewer for this suggestion. We have replaced all RPM values with relative centrifugal force (×*g*) values calculated based on the rotor radius of our centrifuge. Specifically, 3,000 rpm corresponds to approximately 800 ×*g* and 10,000 rpm correspond to approximately 9,000 ×*g*.

Figure 2 legend: can the authors add to the legend the rationale for inclusion of essential genes without confirmation in fitness assay as well as the "not tested" strains in the figure.

>>We thank the reviewer for this important question. We have expanded the Figure 2 legend to clarify the rationale for including essential genes and "not tested" mutants in the pathway analyses. Our objective was to provide a systems-level overview of IE-associated fitness pathways, including genes that could not be experimentally validated because mutants were either unrecoverable from the archived library or found to be essential for viability.

Including these genes provides important biological context and allows interpretation of experimentally validated mutants within complete metabolic and functional pathways. For example, within the CoA biosynthesis pathway that have a total of five coA synthesis genes, all three non-essential genes, *coaA*, *coaB* and *coaC* were identified as IE-related fitness factors. Inclusion of *coaD* and *coaE*, which are predicted essential genes and therefore unavailable for testing, allowed the pathway to be interpreted as an integrated metabolic module associated with IE adaptation. We have clarified this rationale in the revised figure legend.

"Known essential genes within each pathway, as well as mutants unrecovered from the archived library (designated as "not tested), are included. These components are retained within the networks to provide complete biological and metabolic context, allowing experimentally validated

fitness factors to be interpreted within fully integrated functional pathways." These non-tested components are retained within the networks to provide complete biological and metabolic context, allowing experimentally validated fitness factors to be interpreted within fully integrated functional pathways (for example, mapping the essential genes *coaD* and *coaE* alongside the validated fitness determinants *coaA*, *coaB*, and *coaC*)."

Figure 3F: can the authors clarify in the figure legend if these are CFU from competitive index infections or from single strain infections.

>> We thank the reviewer for this important question. The CFU values shown in Figure 3F represent total bacterial recovery from infections in which wild-type and coaC mutant strains were co-inoculated into individual animals at a 1:1 ratio in the initial inoculum. We have clarified this information in the revised figure legend. – in the legend" CFU values represent total bacterial recovery from infections in which wild-type and *coaC* mutant strains were co-inoculated into individual animals at a 1:1 ratio in the initial inoculum."

Figure 4 and 5D-I: the exact same genetic information should be included in each image
>> We thank the reviewer for this constructive suggestion to improve the clarity and consistency of our figures. We have revised both figures to ensure that the genetic and organismal background is explicitly clear across all panels. Specifically: Figure 4 (Panels A–D): The species and strain designation "S. mutans UA159" has been added to the right side of the header bar for every sequencing track. Figure 5 (Panels D–I): The background designation "S. sanguinis SK36" has been added to the right side of the header bar for every sequencing track, and the background strain has been explicitly noted within the Panel D inset.

Figure 4: the labeling within the inset text size variation leads to truncations of gene names making it unclear that the same region is shown in A-D.
>> We thank the reviewer for identifying this formatting issue. We have completely redesigned the zoomed-in genomic insets in Figure 4 to ensure visual continuity. Specifically, we standardized the horizontal zoom scale, window width, and font size across the red inset boxes for Panels A, B, C, and D. By bringing all panels to the identical zoom scale, the typographical truncation of the gene names inside the yellow arrows has been eliminated, making the text fully legible. Furthermore, this uniform scaling ensures that the locus tags (SMU255 through SMU259) align in a perfect vertical layout down the page, making it immediately clear to the reader that the exact same genomic region is being depicted across all four conditions. Finally, we cleaned up the margins of the panels to remove any stray background text artifacts.

Figure 5C: this does not clearly show an attenuation of the non-evolved isolate, could quantitative data be provided instead?

>>We thank the reviewer for this suggestion. To provide quantitative support for the observed phenotypes, we added quantitative measurements of CFU after 20 hr growth to our revised Figure 5C.

Reviewer #3: 1. The manuscript occasionally uses "virulence" when the data more directly reflect infective endocarditis (IE)-associated fitness. The authors do account for this to some extent by examining laboratory growth and validating infection-specific defects; however, the screen mainly identifies genes required for bacteria to survive, grow, compete, and adapt within cardiac vegetations rather than classical virulence mechanisms such as toxin production or immune damage. Many of the identified pathways therefore appear to function more as niche adaptation

or IE-associated fitness determinants. I therefore suggest that terms such as "IE-associated fitness factors" or "IE-associated niche factors" more consistently throughout the manuscript, as this would improve clarity and better reflect the biological implications of the findings in this work.

>> We thank the reviewer for this important conceptual suggestion. Throughout the revised manuscript, we have replaced the term "virulence factors" with "IE-associated fitness factors" or related terminology where appropriate to more accurately reflect that the identified genes primarily contribute to bacterial survival, adaptation, and persistence within cardiac vegetations rather than classical toxin-mediated virulence mechanisms.

2. I would suggest the authors not to use the term "redefine IE" but really the work provides a genome-wide map of bacterial niche-requirement for streptococcal IE.

>> We thank the reviewer for this constructive suggestion. We agree that describing the work as providing a genome-wide map of niche-specific requirements is more accurate and grounded. We have revised the final sentence of the Abstract (as well as similar phrasing in the main text) to replace "redefine" with language that emphasizes the comprehensive mapping of these streptococcal fitness networks. In the abstract, "Together, these findings provide a comprehensive, genome-wide map of the bacterial niche-requirements for streptococcal infective endocarditis."

3. The authors made great effort to control bottleneck effect, but this remained to be effectively defined as it took some time for me to relate what the authors really meant. I think it is worthy of additional explanations that it is essentially a severe reduction in population diversity during infection establishment, in that with a high variation library, some mutants may disappear simply due to stochastic loss, sampleing effect and low initial representation rather than true biological fitness defects. This made it easier to understand why the authors make good efforts to experimentally judge the best inocula pool size (lines 134-150).

>>We thank the reviewer for this insightful suggestion. We agree that the concept of the infection bottleneck was not sufficiently defined in the original manuscript. We have revised the Results sections to more clearly explain that the bottleneck effect refers to a severe reduction in mutant population diversity during infection establishment, in which some mutants may be lost because of stochastic sampling effects or low initial representation rather than true biological fitness defects. We have also clarified that this concern motivated our experimental optimization of inoculum pool size to minimize stochastic mutant loss and improve the accuracy of IE-associated fitness identification. "A major challenge in mixed mutant library analysis during infection is the occurrence of severe population bottlenecks during host colonization. In highly diverse mutant libraries, some mutants may be randomly lost because of stochastic sampling effects or low initial abundance rather than genuine fitness defects, potentially leading to false-positive identification of conditionally important genes. To minimize these effects, we experimentally optimized the inoculum pool size to maximize mutant representation and preserve library diversity during IE establishment."

4. In line 239 on rhamnan synthesis, I think it should be rml instead of rlm genes. Please also verify the order of the genes – they appeared in blocks, but the alphabet is normally referred to their enzymatic order in synthesising dTDP-rhamnose.

>> We thank the reviewer for identifying this error. We have corrected "rlm" to "rml" throughout the manuscript and verified the gene order according to the established enzymatic pathway for dTDP-rhamnose biosynthesis.

5. In line 236 and line 247, on the requirement for genes involvement in the shikimate and serine pathway during IE. The authors may relate to the broader downstream metabolic roles of these pathways beyond amino acid biosynthesis alone. In particular, chorismate- and serine-associated metabolism contribute to folate, one-carbon, nucleotide, and quinone biosynthetic pathways that may be important for metabolic robustness, redox balance, and adaptation within the host environment.

>> We thank the reviewer for this excellent suggestion. We agree that the importance of the shikimate and serine pathways during IE likely stems from their roles in global metabolic orchestration rather than amino acid production alone. We have updated the Discussion section to explicitly link these pathways to their downstream contributions to folate biosynthesis, one-carbon metabolism, nucleotide and quinone production, redox homeostasis, and general in-host fitness.

In the 4th paragraph of Discussion "Notably, the requirement for the shikimate and serine pathways likely extends beyond amino acid biosynthesis alone; chorismate- and serine-associated metabolism drive downstream folate, one-carbon, nucleotide, and quinone biosynthesis crucial for metabolic robustness, redox balance, and host adaptation."

6. In Fig 3, the color codes used may not be very friendly to a partial colour-blind, especially the shikimate vs rml colours (please either use an alternative color scheme or simply label the boxes).

>> We thank the reviewer for this recommendation. We have revised the figure by labeling the boxes to improve figure accessibility and readability for color-blind readers.

7. Shield et al (ref 42) noted the coaC and shikimate pathway genes as essential in strain UA159. This may be strain variation, but also indicate that severe compromise can confound interpretation of transposon essentiality studies, see doi: 10.1128/spectrum.01303-25 for a relevant discussion. The latter is supported by the Fig 3E in this work.

>> We thank the reviewer for pointing this out, and we entirely agree. Severe fitness compromise can indeed confound transposon essentiality dynamics, blending the lines between true essentiality and conditional fitness defects. We have updated our Discussion section to address this technical consideration: " While Shield et al. previously classified *coaC* and the shikimate pathway as strictly essential in *S. mutans* UA159 (PMID: 29435491), our identification of these as conditional fitness factors may reflect the challenge of distinguishing true genetic essentiality from severe fitness compromise in high-density transposon libraries (PMID: 40810554). Here, whole-genome sequencing verified our target deletion genotypes against the UA159 reference NC_003450 (Figure 4; raw reads deposited under GenBank: PRJNA1431959)."

3. Additional suggestions by the Journal

Figure resubmission:

While revising your submission, we strongly recommend that you use PLOS's NAAS tool (https://ngplosjournals.pagemajik.ai/artanalysis) to test your figure files. NAAS can convert your figure files to the TIFF file type and meet basic requirements (such as print size, resolution), or provide you with a report on issues that do not meet our requirements and that NAAS cannot fix. After uploading your figures to PLOS's NAAS tool - https://ngplosjournals.pagemajik.ai/artanalysis, NAAS will process the files provided and display

the results in the "Uploaded Files" section of the page as the processing is complete. If the uploaded figures meet our requirements (or NAAS is able to fix the files to meet our requirements), the figure will be marked as "fixed" above. If NAAS is unable to fix the files, a red "failed" label will appear above. When NAAS has confirmed that the figure files meet our requirements, please download the file via the download option, and include these NAAS processed figure files when submitting your revised manuscript.

>>Thank you for the guidance. We have fixed the figures using PLOS's NAAS tool.

Reproducibility:

To enhance the reproducibility of your results, we recommend that authors of applicable studies deposit laboratory protocols in protocols.io, where a protocol can be assigned its own identifier (DOI) such that it can be cited independently in the future. Additionally, PLOS ONE offers an option to publish peer-reviewed clinical study protocols. Read more information on sharing protocols at https://plos.org/protocols?utm_medium=editorial-email&utm_source=authorletters&utm_campaign=protocols

>>Thank you for the opportunity. We will consider working on a manuscript to deposit laboratory protocols in protocols.io.
