# Supplementary material for: A genome-wide in vivo screen reveals fitness pathways required for streptococcal infective endocarditis
Source: PLoS Pathog. 2026 Aug 17;22(8):e1014156. doi: 10.1371/journal.ppat.1014156 (PMC13506083; doi:10.1371/journal.ppat.1014156)
Supplement: Supplementary file 2 [file ppat.1014156.s021.pdf]

We sincerely thank Dr. Sam Manna, Dr. Helena Boshoff, the Academic Editor, and the Editorial Team at PLOS Pathogens for their thoughtful evaluation of our manuscript (PPATHOGENS-D-26-00900R1) and for the opportunity to submit a revised version. We appreciate the constructive feedback, which has helped improve the manuscript. Below, we provide a point-by-point response to the editor's comments and summarize the revisions made.

** A letter that responds to each point raised by the editor and reviewer(s). You should upload this letter as a separate file labeled 'Response to Reviewers'. This file does not need to include responses to any formatting updates and technical items listed in the 'Journal Requirements' section below.*
** A marked-up copy of your manuscript that highlights changes made to the original version. You should upload this as a separate file labeled 'Revised Manuscript with Track Changes'.*
** An unmarked version of your revised paper without tracked changes. You should upload this as a separate file labeled 'Manuscript'.*

>>We have prepared and uploaded all three required files:

Response to Reviewers – Point-by-point responses addressing the Academic Editor's comments.

Revised Manuscript with Track Changes – A marked-up version highlighting all revisions, including updates to the Discussion section.

Manuscript – A clean version of the revised manuscript without tracked changes.

If you would like to make changes to your financial disclosure, competing interests statement, or data availability statement, please make these updates within the submission form at the time of resubmission. Guidelines for resubmitting your figure files are available below the reviewer comments at the end of this letter.

>> Thank you for the guidance. We have updated the Financial Disclosure statement in the submission system in accordance with the journal's requirements.

As the corresponding author, your ORCID iD is verified in the submission system and will appear in the published article. PLOS supports the use of ORCID, and we encourage all coauthors to register for an ORCID iD and use it as well. Please encourage your coauthors to verify their ORCID iD within the submission system before final acceptance, as unverified ORCID iDs will not appear in the published article. Only the individual author can complete the verification step; PLOS staff cannot verify ORCID iDs on behalf of authors.

>> Thank you for the reminder. Requests have been sent to all co-authors to verify their ORCID iDs in the submission system.

Additional Editor Comments (if provided):*Although most reviewer comments have been appropriately addressed, the reviewer comments around the fitness/growth defects observed in vitro as well as in IE needs to be more strongly acknowledged as a limitation of the study in the discussion. A more severe growth defect in IE compared with in vitro does not necessarily mean that genes are important or involved in IE. Please acknowledge this caveat in the discussion by noting caution in interpretation of the findings in of specific mutants in IE when similar growth defects are observed in vitro.*

>>We thank the Academic Editor for this important suggestion and fully agree that this limitation should be more explicitly acknowledged. To address this concern, we have added a new paragraph to the Discussion (first paragraph) emphasizing that in vivo fitness defects should be interpreted cautiously when similar growth defects are also observed under in vitro conditions. The revised text reads:

"Fourth, a limitation of this study is the interpretation of in vivo fitness defects for mutants that also exhibit growth or fitness defects in vitro (Figure 3A–E). Although several mutants displayed defects under both conditions, reduced fitness in the rabbit IE model does not necessarily indicate that these genes play a specific role in IE pathogenesis. Instead, impaired fitness in vivo may reflect general physiological or growth defects that compromise bacterial survival and proliferation across multiple environments. Consequently, the contribution of genes exhibiting concurrent in vitro and in vivo phenotypes should be interpreted with caution, as the in vivo phenotype alone cannot distinguish between general fitness requirements and infection-specific functions. Additional mechanistic studies will be required to define the specific roles of these genes during IE."

Journal Requirements: If the reviewer comments include a recommendation to cite specific previously published works, please review and evaluate these publications to determine whether they are relevant and should be cited. There is no requirement to cite these works unless the editor has indicated otherwise. 1) Please amend your detailed Financial Disclosure statement. This is published with the article. It must therefore be completed in full sentences and contain the exact wording you wish to be published.

Updated per journal requirements. i) State what role the funders took in the study. If the funders had no role in your study, please state: "The funders had no role in study design, data collection and analysis, decision to publish, or preparation of the manuscript."
ii) If any authors received a salary from any of your funders, please state which authors and which funders. If you did not receive any funding for this study, please simply state: u201cThe authors received no specific funding for this work.u201d

>>We have revised the Financial Disclosure statement in the submission system to comply with PLOS Pathogens requirements. The updated statement reads: " This work was funded by a grant from the National Institutes of Health R01DE030121 (P.X. and T.K.). P.X. was supported by the CCTR Endowment Fund. T.K. was supported by R21DE034103. L.B. was supported by R03DE034511. K.M.T. is a member of the VCU Massey Comprehensive Cancer Center Bioinformatics Shared Resource, which receives funding from NIH-NCI Cancer Center Support Grant P30 CA016059. The funders had no role in study design, data collection and analysis, decision to publish, or preparation of the manuscript."

Reviewers' Comments: *[NOTE: If reviewer comments were submitted as an attachment file, they will be attached to this email and accessible via the submission site. Please log into your account, locate the manuscript record, and check for the action link "View Attachments". If this link does not appear, there are no attachment files.]*
>> We thank the reviewers for their constructive comments during the previous round of review, which helped strengthen the manuscript. No additional reviewer comments were provided in this decision letter.

Figure resubmission: *While revising your submission, we strongly recommend that you use PLOS's NAAS tool (https://ngplosjournals.pagemajik.ai/artanalysis) to test your figure files.*

NAAS can convert your figure files to the TIFF file type and meet basic requirements (such as print size, resolution), or provide you with a report on issues that do not meet our requirements and that NAAS cannot fix. After uploading your figures to PLOS's NAAS tool - https://ngplosjournals.pagemajik.ai/artanalysis, NAAS will process the files provided and display the results in the "Uploaded Files" section of the page as the processing is complete. If the uploaded figures meet our requirements (or NAAS is able to fix the files to meet our requirements), the figure will be marked as "fixed" above. If NAAS is unable to fix the files, a red "failed" label will appear above. When NAAS has confirmed that the figure files meet our requirements, please download the file via the download option, and include these NAAS processed figure files when submitting your revised manuscript

>> Thank you for the recommendation. We processed all figure files using the PLOS NAAS tool and have uploaded the validated figures with our revised submission.

Reproducibility:*To enhance the reproducibility of your results, we recommend that authors of applicable studies deposit laboratory protocols in protocols.io, where a protocol can be assigned its own identifier (DOI) such that it can be cited independently in the future. Additionally, PLOS ONE offers an option to publish peer-reviewed clinical study protocols. Read more information on sharing protocols at https://plos.org/protocols?utm_medium=editorial-email&utm_source=authorletters&utm_campaign=protocols*

>>We appreciate the Academic Editor's careful evaluation and believe that the revisions made have fully addressed the outstanding concerns. We thank the Editorial Team for their consideration and look forward to your final decision.
