# Supplementary material for: A genome-wide in vivo screen reveals fitness pathways required for streptococcal infective endocarditis
Source: PLoS Pathog. 2026 Aug 17;22(8):e1014156. doi: 10.1371/journal.ppat.1014156 (PMC13506083; doi:10.1371/journal.ppat.1014156)
Supplement: Peer review history

# Peer review: Decision Letter 0

**DOI:** 10.1371/journal.ppat.1014156.r001

**Authors:** Helena Boshoff, Sam Manna

22 May 2026

A genome-wide in vivo screen reveals fitness pathways required for streptococcal infective endocarditis

Dear Dr. Bao,

Thank you for submitting your manuscript to PLOS Pathogens. After careful consideration, we feel that it has merit but does not fully meet PLOS Pathogens's publication criteria as it currently stands. Therefore, we invite you to submit a revised version of the manuscript that addresses the points raised during the review process.

Please submit your revised manuscript by Jul 21 2026 11:59PM. If you will need more time than this to complete your revisions, please reply to this message or contact the journal office at plospathogens@plos.org. When you're ready to submit your revision, log on to https://www.editorialmanager.com/ppathogens/ and select the 'Submissions Needing Revision' folder to locate your manuscript file.

Please include the following items when submitting your revised manuscript:

* A letter that responds to each point raised by the editor and reviewer(s). You should upload this letter as a separate file labeled 'Response to Reviewers'. This file does not need to include responses to any formatting updates and technical items listed in the 'Journal Requirements' section below.

* A marked-up copy of your manuscript that highlights changes made to the original version. You should upload this as a separate file labeled 'Revised Manuscript with Track Changes'.

* An unmarked version of your revised paper without tracked changes. You should upload this as a separate file labeled 'Manuscript'.

We look forward to receiving your revised manuscript.

Kind regards,

Sam Manna, PhD

Academic Editor

PLOS Pathogens

Helena Boshoff

Section Editor

Editor-in-Chief

PLOS Pathogens

Michael Malim

Editor-in-Chief

PLOS Pathogens

orcid.org/0000-0002-7699-2064

**Additional Editor Comments:**

Please pay particular attention to the points raised by multiple reviewers about differentiating whether the genes identified in your study are actual infective endocarditis virulence determinants or simply important for growth and the requirement for an expanded bioinformatic analysis for these genes in both IE and non-IE causing species.

**Journal Requirements:**

1) Please ensure that the CRediT author contributions listed for every co-author are completed accurately and in full.

At this stage, the following Authors/Authors require contributions: Liang Bao, Jennifer Bradley, Vysakh Anandan, Katarzyna Tyc, Zan Zhu, Josephina Anna Vossen, Valery francine Assi, Jasmine Benbei, Nicai Zollar, Todd Kitten, and Ping Xu. Please ensure that the full contributions of each author are acknowledged in the "Add/Edit/Remove Authors" section of our submission form.

The list of CRediT author contributions may be found here: https://journals.plos.org/plospathogens/s/authorship#loc-author-contributions

2) We ask that a manuscript source file is provided at Revision. Please upload your manuscript file as a .doc, .docx, .rtf or .tex. If you are providing a .tex file, please upload it under the item type u2018LaTeX Source Fileu2019 and leave your .pdf version as the item type u2018Manuscriptu2019.

3) Please provide an Author Summary. This should appear in your manuscript between the Abstract (if applicable) and the Introduction, and should be 150-200 words long. The aim should be to make your findings accessible to a wide audience that includes both scientists and non-scientists. Sample summaries can be found on our website under Submission Guidelines:

https://journals.plos.org/plospathogens/s/submission-guidelines#loc-parts-of-a-submission

4) Please upload all main figures as separate Figure files in .tif or .eps format. For more information about how to convert and format your figure files please see our guidelines:

https://journals.plos.org/plospathogens/s/figures

5) We have noticed that you have uploaded Supporting Information files, but you have not included a list of legends. Please add a full list of legends for your Supporting Information files after the references list.

6) Some material included in your submission may be copyrighted. According to PLOS's copyright policy, authors who use figures or other material (e.g., graphics, clipart, maps) from another author or copyright holder must demonstrate or obtain permission to publish this material under the Creative Commons Attribution 4.0 International (CC BY 4.0) License used by PLOS journals. Please closely review the details of PLOS's copyright requirements here: <a data-cke-saved-href=" https://journals.plos.org/plospathogens/s/licenses-and-copyright" href=" https://journals.plos.org/plospathogens/s/licenses-and-copyright">PLOS Licenses and Copyright</a>. If you need to request permissions from a copyright holder, you may use the&nbsp;<a data-cke-saved-href=" https://storage.googleapis.com/plos-published-prod/c4ff/Content%20Copyright%20Permission%20Form.pdf?X-Goog-Algorithm=GOOG4-RSA-SHA256&X-Goog-Credential=wombat-sa%40plos-prod.iam.gserviceaccount.com%2F20220817%2Fauto%2Fstorage%2Fgoog4_request&X-Goog-Date=20220817T210608Z&X-Goog-Expires=86400&X-Goog-SignedHeaders=host&X-Goog-Signature=cd9dc868773378f7033b9f8cfb91430167d6275df90bce01342a454f36512530b64f24736fbf68c4fd96da74d663b6ee95b3420bded8fb0e833af2390b896b8706643b476d7f532408921b60576d65b60617070900fd2bcd28eec4cd32fefcf007d3655df17fcc91023329a28479ce0d1c5758174eedcc9e3c1788c5f7dd8523f3ddd871e02c998a20f7dc78ec00f313d4b5a78ba2037a6c7ef0a05dc29817da9368065460c8d8446659f35f0458fefabd3101eb7f926e9cb7a0b53e7bbe47aaed3e0ba52f9af9c2b8f6d7d3a8871b205fc4cbe97384ed37c7604fd7eecfedccf49cba12820b2e3b649541cefde3103cd7d72731b3e8b0248439b4cba691ca8a" href=" https://storage.googleapis.com/plos-published-prod/c4ff/Content%20Copyright%20Permission%20Form.pdf?X-Goog-Algorithm=GOOG4-RSA-SHA256&X-Goog-Credential=wombat-sa%40plos-prod.iam.gserviceaccount.com%2F20220817%2Fauto%2Fstorage%2Fgoog4_request&X-Goog-Date=20220817T210608Z&X-Goog-Expires=86400&X-Goog-SignedHeaders=host&X-Goog-Signature=cd9dc868773378f7033b9f8cfb91430167d6275df90bce01342a454f36512530b64f24736fbf68c4fd96da74d663b6ee95b3420bded8fb0e833af2390b896b8706643b476d7f532408921b60576d65b60617070900fd2bcd28eec4cd32fefcf007d3655df17fcc91023329a28479ce0d1c5758174eedcc9e3c1788c5f7dd8523f3ddd871e02c998a20f7dc78ec00f313d4b5a78ba2037a6c7ef0a05dc29817da9368065460c8d8446659f35f0458fefabd3101eb7f926e9cb7a0b53e7bbe47aaed3e0ba52f9af9c2b8f6d7d3a8871b205fc4cbe97384ed37c7604fd7eecfedccf49cba12820b2e3b649541cefde3103cd7d72731b3e8b0248439b4cba691ca8a">PLOS Content Copyright Permission&nbsp;Form</a>

Please respond directly to this email and provide any known details concerning your material's license terms and permissions required for reuse, even if you have not yet obtained copyright permissions or are unsure of your material's copyright compatibility. Once you have responded and addressed all other outstanding technical requirements, you may resubmit your manuscript within Editorial Manager.

Potential Copyright Issues:

- Figure 1:  Please confirm whether you drew the images / clip-art within the figure panels by hand. If you did not draw the images, please provide (a) a link to the source of the images or icons and their license / terms of use; or (b) written permission from the copyright holder to publish the images or icons under our CC BY 4.0 license. Alternatively, you may replace the images with open source alternatives. See these open source resources you may use to replace images / clip-art:

- https://commons.wikimedia.org

- https://openclipart.org/.

7) Please amend your detailed Financial Disclosure statement. This is published with the article. It must therefore be completed in full sentences and contain the exact wording you wish to be published.

- State what role the funders took in the study. If the funders had no role in your study, please state: "The funders had no role in study design, data collection and analysis, decision to publish, or preparation of the manuscript.".

If you did not receive any funding for this study, please simply state: u201cThe authors received no specific funding for this work.u201d

**Reviewers' Comments:**

Reviewer's Responses to Questions

**Part I - Summary**

Please use this section to discuss strengths/weaknesses of study, novelty/significance, general execution and scholarship.

Reviewer #1: This manuscript describes the search for a nonbiased list of genes that contribute to infective endocarditis (IE) caused by oral streptococci. This is a medically complex disease that is difficult to treat. The discovery of novel targets for therapies would be welcome. Previous work has focused on this problem is clearly recognized by the authors. They then openly discuss the many possible bottlenecks and limitations of the many studies using this validated animal model and develop a strategy to more broadly ask the question of how the endocardial lesions are colonized and infection progresses.

The approach is intriguing and the results indicate success in developing a much wider genetic understanding of the fitness factors that promote disease than previously attained. The study initially uses strep sanguinis and is then repeated in the similar pathogen strep mutans. The genes are also compared across other pathogens that cause endocarditis but in more limited settings. The fact the there is considerable overlap lends support to their conclusions. They also look for compensatory mutations that arise during passage of knock out mutants. This is a clever approach to further support validation.

This study is well thought out and the conclusions are robust, rigorously tested and, importantly, do not go beyond the data.

Reviewer #2: Bao et al present a genetic screen to identify genetic determinants of S. sanguinis infective endocarditis in an animal model. The strategy of repeat testing of mutants in different pools adds robustness to the study. An additional strength is the confirmation of these putative virulence factors in an S. mutans IE model. The attempt to isolate the mechanism of action for these putative virulence factors by comparing growth in rich media and in serum to the in vivo colonization defects was not as well supported, as many of the putative IE virulence factors had reduced growth in rich media, suggesting that they may be generally attenuated, instead of specifically attenuated in the IE model. The attempt to identify suppressor mutants through experimental evolution was a strength of the study, however the failure to test the experimentally evolved strains in the IE model was a weakness. Further, while the authors suggest the importance of the identified factors as being important for infective endocarditis, the experiments do not adequately differentiate between the ability for the bacteria to survive in the blood versus the ability for the bacteria to colonize the endocardium. Additionally, the authors discuss the conservation of these IE virulence factors in many IE pathogens as support for these being genetic determinants of IE pathogenesis, however, this argument is weakened by the failure to show that these genetic determinants are absent from bacterial pathogens that do not cause endocarditis.

Reviewer #3: This is a strong and technically ambitious manuscript presenting the first genome-wide in vivo fitness analysis of Streptococcus sanguinis IE using a vertebrate model. The study provides a substantial dataset that offers a coherent physiological insight into the pathways required for the pathogen to establish IE. While I appreciate the technical rigor and experimental care the authors presented in this story, I do have several minor comments I wish the authors would consider, and mostly regarding conceptual framing and resolving clarities.

**********

**Part II – Major Issues: Key Experiments Required for Acceptance**

Please use this section to detail the key new experiments or modifications of existing experiments that should be absolutely required to validate study conclusions.

Generally, there should be no more than 3 such required experiments or major modifications for a "Major Revision" recommendation. If more than 3 experiments are necessary to validate the study conclusions, then you are encouraged to recommend "Reject".

Reviewer #1: None

Reviewer #2: The experiments as designed cannot differentiate between survival and growth in the bloodstream versus ability to colonize the endocardium. If the authors could take peripheral blood from the infected animals at the time of tissue harvest and compare relative frequency of mutants in the blood versus in the vegetation, that would support that the identified virulence factors are specific to IE and not general sepsis virulence factors. Alternatively, to reduce animal numbers, growth of libraries in whole blood (rabbit, or even human to increase the relevance of the model to human disease) would be acceptable.

As the putative IE virulence factors are involved in essential functions, it is not surprising that there is a high degree of conservation with other taxa. The authors assert that finding these putative virulence factors in many bacterial strains that cause IE supports the role of these gene products in IE. However, unless they authors could show a lack of conservation of these factors in bacteria that do not cause IE, this conservation is merely coincidental.

Lines 231-252: the in vitro growth defects seen do not support the roles of these as IE virulence factors, instead they seem to cause a general attenuation in growth.

The details of generation of the S. mutans deletion strains are not present in the materials and methods section, and should be provided.

The differences in in vitro growth attenuation between the S. sanguinis and the S. mutans shikimate mutants does not support the overall conclusions of the story. I suggest focusing on the coaC which had similar phenotypes in both species to better support the overall conclusions of this paper.

While the experimental evolution for both the S. mutans shikimate mutants and the S. sanguinis CoA mutants demonstrate restoration of in vitro growth, to support the conclusion of these suppressor mutants being important in IE, the restoration of the ability to colonize cardiac vegetations is necessary. The evolved isolates should be tested in the IE model.

Reviewer #3: I don't think additional experiments are required for this work.

**********

**Part III – Minor Issues: Editorial and Data Presentation Modifications**

Please use this section for editorial suggestions as well as relatively minor modifications of existing data that would enhance clarity.

Reviewer #1: One question arises out of curiosity and could be more thoroughly discussed. The comparison of genomes of a variety of species known to cause endocarditis yields similarities. Does the reverse also hold true? Do species that rarely cause endocarditis not have the right “equipment”? or is this disease reliant on flexibility of changing metabolism as shown by the compensatory mutation results rather than a specific gene set? Would a mutant in transformation pathway so well known for these streps be a key part of the picture?

Reviewer #2: Line 32: As antibiotic discovery is not tested in this study, this statement could be removed from the abstract.

Line 70: Could the authors define what cardiac conditions elevate the risk of endocarditis? Also could the authors mention the elevated risk in injection drug users.

Lines 143 and 145: is this the wild type strain or the ssa_0169 mutant?

Line 180: Why were these mutants absent from the input library? Are they essential genes?

Line 224: is 39C typical of human infections or only the rabbit model?

Line 248: a citation of the availability of serine in cardiac vegetations is necessary.

Line 270: is SMU277 a homolog of ssa_0169? Or does it just have a similar phenotype?

Paragraph beginning line 406: Leveraging these putative virulence factors as a target for design of rationale therapeutics opens the concern for toxicity to the microbiome, which is not addressed. The authors state high conservation of these putative genes in Streptococci and Enterococci, but these taxa are also common colonizers of the GI tract, and the authors should address if these candidate genes are present in GI colonizing strains.

Lines 462 and 482: could the authors report centrifugation conditions as xg rather than RPM which can vary from unit to unit.

Figure 2 legend: can the authors add to the legend the rationale for inclusion of essential genes without confirmation in fitness assay as well as the “not tested” strains in the figure.

Figure 3F: can the authors clarify in the figure legend if these are CFU from competitive index infections or from single strain infections.

Figure 4 and 5D-I: the exact same genetic information should be included in each image

Figure 4: the labeling within the inset text size variation leads to truncations of gene names making it unclear that the same region is shown in A-D.

Figure 5C: this does not clearly show an attenuation of the non-evolved isolate, could quantitative data be provided instead?

Reviewer #3: 1. The manuscript occasionally uses “virulence” when the data more directly reflect infective endocarditis (IE)-associated fitness. The authors do account for this to some extent by examining laboratory growth and validating infection-specific defects; however, the screen mainly identifies genes required for bacteria to survive, grow, compete, and adapt within cardiac vegetations rather than classical virulence mechanisms such as toxin production or immune damage. Many of the identified pathways therefore appear to function more as niche adaptation or IE-associated fitness determinants. I therefore suggest that terms such as “IE-associated fitness factors” or “IE-associated niche factors” more consistently throughout the manuscript, as this would improve clarity and better reflect the biological implications of the findings in this work.

2. I would suggest the authors not to use the term “redefine IE” but really the work provides a genome-wide map of bacterial niche-requirement for streptococcal IE.

3. The authors made great effort to control bottleneck effect, but this remained to be effectively defined as it took some time for me to relate what the authors really meant. I think it is worthy of additional explanations that it is essentially a severe reduction in population diversity during infection establishment, in that with a high variation library, some mutants may disappear simply due to stochastic loss, sampleing effect and low initial representation rather than true biological fitness defects. This made it easier to understand why the authors make good efforts to experimentally judge the best inocula pool size (lines 134-150).

4. In line 239 on rhamnan synthesis, I think it should be rml instead of rlm genes. Please also verify the order of the genes – they appeared in blocks, but the alphabet is normally referred to their enzymatic order in synthesising dTDP-rhamnose.

5. In line 236 and line 247, on the requirement for genes involvement in the shikimate and serine pathway during IE. The authors may relate to the broader downstream metabolic roles of these pathways beyond amino acid biosynthesis alone. In particular, chorismate- and serine-associated metabolism contribute to folate, one-carbon, nucleotide, and quinone biosynthetic pathways that may be important for metabolic robustness, redox balance, and adaptation within the host environment.

6. In Fig 3, the color codes used may not be very friendly to a partial colour-blind, especially the shikimate vs rml colours (please either use an alternative color scheme or simply label the boxes).

7. Shield et al (ref 42) noted the coaC and shikimate pathway genes as essential in strain UA159. This may be strain variation, but also indicate that severe compromise can confound interpretation of transposon essentiality studies, see doi: 10.1128/spectrum.01303-25 for a relevant discussion. The latter is supported by the Fig 3E in this work.

**********

PLOS authors have the option to publish the peer review history of their article (what does this mean?). If published, this will include your full peer review and any attached files.

If you choose “no”, your identity will remain anonymous but your review may still be made public.

**Do you want your identity to be public for this peer review?**  For information about this choice, including consent withdrawal, please see our Privacy Policy.

Reviewer #1: **Yes:** Elaine Tuomanen

Reviewer #2: No

Reviewer #3: No

[NOTE: If reviewer comments were submitted as an attachment file, they will be attached to this email and accessible via the submission site. Please log into your account, locate the manuscript record, and check for the action link "View Attachments". If this link does not appear, there are no attachment files.]

**Figure resubmission:**

**Reproducibility:**

To enhance the reproducibility of your results, we recommend that authors of applicable studies deposit laboratory protocols in protocols.io, where a protocol can be assigned its own identifier (DOI) such that it can be cited independently in the future. Additionally, PLOS ONE offers an option to publish peer-reviewed clinical study protocols. Read more information on sharing protocols at https://plos.org/protocols?utm_medium=editorial-email&utm_source=authorletters&utm_campaign=protocols

# Author comment: Author response to Decision Letter 1

**DOI:** 10.1371/journal.ppat.1014156.r002

22 Jul 2026

Attachment Submitted filename: PLOS Path. reply to Reviewers comments-resubmit_07192026.pdf

# Editor report: Decision Letter 1

**DOI:** 10.1371/journal.ppat.1014156.r003

**Authors:** Helena Boshoff, Sam Manna

29 Jul 2026

PPATHOGENS-D-26-00900R1

A genome-wide in vivo screen reveals fitness pathways required for streptococcal infective endocarditis

PLOS Pathogens

Dear Dr. Bao,

Thank you for submitting your manuscript to PLOS Pathogens. After careful consideration, we feel that it has merit but does not fully meet PLOS Pathogens's publication criteria as it currently stands. Therefore, we invite you to submit a revised version of the manuscript that addresses the points raised during the review process.

Please submit your revised manuscript by Sep 27 2026 11:59PM. If you will need more time than this to complete your revisions, please reply to this message or contact the journal office at plospathogens@plos.org. When you're ready to submit your revision, log on to https://www.editorialmanager.com/ppathogens/ and select the 'Submissions Needing Revision' folder to locate your manuscript file.

Please include the following items when submitting your revised manuscript:

* A letter that responds to each point raised by the editor and reviewer(s). You should upload this letter as a separate file labeled 'Response to Reviewers'. This file does not need to include responses to any formatting updates and technical items listed in the 'Journal Requirements' section below.

* A marked-up copy of your manuscript that highlights changes made to the original version. You should upload this as a separate file labeled 'Revised Manuscript with Track Changes'.

* An unmarked version of your revised paper without tracked changes. You should upload this as a separate file labeled 'Manuscript'.

If you would like to make changes to your financial disclosure, competing interests statement, or data availability statement, please make these updates within the submission form at the time of resubmission. Guidelines for resubmitting your figure files are available below the reviewer comments at the end of this letter.

As the corresponding author, your ORCID iD is verified in the submission system and will appear in the published article. PLOS supports the use of ORCID, and we encourage all coauthors to register for an ORCID iD and use it as well. Please encourage your coauthors to verify their ORCID iD within the submission system before final acceptance, as unverified ORCID iDs will not appear in the published article. Only the individual author can complete the verification step; PLOS staff cannot verify ORCID iDs on behalf of authors.

We look forward to receiving your revised manuscript.

Kind regards,

Sam Manna, PhD

Academic Editor

PLOS Pathogens

Helena Boshoff

Section Editor

PLOS Pathogens

Sumita Bhaduri-McIntosh

Editor-in-Chief

PLOS Pathogens

orcid.org/0000-0003-2946-9497

Michael Malim

Editor-in-Chief

PLOS Pathogens

orcid.org/0000-0002-7699-2064

**Additional Editor Comments (if provided):**

Although most reviewer comments have been appropriately addressed, the reviewer comments around the fitness/growth defects observed in vitro as well as in IE needs to be more strongly acknowledged as a limitation of the study in the discussion. A more severe growth defect in IE compared with in vitro does not necessarily mean that genes are important or involved in IE. Please acknowledge this caveat in the discussion by noting caution in interpretation of the findings in of specific mutants in IE when similar growth defects are observed in vitro.

**Journal Requirements:**

If the reviewer comments include a recommendation to cite specific previously published works, please review and evaluate these publications to determine whether they are relevant and should be cited. There is no requirement to cite these works unless the editor has indicated otherwise.

1) Please amend your detailed Financial Disclosure statement. This is published with the article. It must therefore be completed in full sentences and contain the exact wording you wish to be published.

i) State what role the funders took in the study. If the funders had no role in your study, please state: "The funders had no role in study design, data collection and analysis, decision to publish, or preparation of the manuscript."

ii) If any authors received a salary from any of your funders, please state which authors and which funders..

If you did not receive any funding for this study, please simply state: u201cThe authors received no specific funding for this work.u201d

**Reviewers' Comments:**

[NOTE: If reviewer comments were submitted as an attachment file, they will be attached to this email and accessible via the submission site. Please log into your account, locate the manuscript record, and check for the action link "View Attachments". If this link does not appear, there are no attachment files.]

**Figure resubmission:**

**Reproducibility:**

To enhance the reproducibility of your results, we recommend that authors of applicable studies deposit laboratory protocols in protocols.io, where a protocol can be assigned its own identifier (DOI) such that it can be cited independently in the future. Additionally, PLOS ONE offers an option to publish peer-reviewed clinical study protocols. Read more information on sharing protocols at https://plos.org/protocols?utm_medium=editorial-email&utm_source=authorletters&utm_campaign=protocols

# Author comment: Author response to Decision Letter 2

**DOI:** 10.1371/journal.ppat.1014156.r004

31 Jul 2026

Attachment Submitted filename: Response to Reviewers_resubmitted07302026.pdf

# Editor report: Decision Letter 2

**DOI:** 10.1371/journal.ppat.1014156.r005

**Authors:** Helena Boshoff, Sam Manna

4 Aug 2026

Dear Dr. Bao,

We are pleased to inform you that your manuscript 'A genome-wide in vivo screen reveals fitness pathways required for streptococcal infective endocarditis' has been provisionally accepted for publication in PLOS Pathogens.

Before your manuscript can be formally accepted you will need to complete some formatting changes, which you will receive in a follow up email. A member of our team will be in touch with a set of requests.

Please note that your manuscript will not be scheduled for publication until you have made the required changes, so a swift response is appreciated.

IMPORTANT: The editorial review process is now complete. PLOS will only permit corrections to spelling, formatting or significant scientific errors from this point onwards. Requests for major changes, or any which affect the scientific understanding of your work, will cause delays to the publication date of your manuscript.

Should you, your institution's press office or the journal office choose to press release your paper, you will automatically be opted out of early publication. We ask that you notify us now if you or your institution is planning to press release the article. All press must be co-ordinated with PLOS.

Thank you again for supporting Open Access publishing; we are looking forward to publishing your work in PLOS Pathogens.

Best regards,

Sam Manna, PhD

Academic Editor

PLOS Pathogens

Helena Boshoff

Section Editor

PLOS Pathogens

Sumita Bhaduri-McIntosh

Editor-in-Chief

PLOS Pathogens

orcid.org/0000-0003-2946-9497

Michael Malim

Editor-in-Chief

PLOS Pathogens

orcid.org/0000-0002-7699-2064

***********************************************************

Reviewer Comments (if any, and for reference):

# Editor report: Acceptance letter

**DOI:** 10.1371/journal.ppat.1014156.r006

**Authors:** Helena Boshoff, Sam Manna

Dear Dr. Bao,

We are delighted to inform you that your manuscript, "A genome-wide in vivo screen reveals fitness pathways required for streptococcal infective endocarditis," has been formally accepted for publication in PLOS Pathogens.

We have now passed your article onto the PLOS Production Department who will complete the rest of the pre-publication process. All authors will receive a confirmation email upon publication.

The corresponding author will soon be receiving a typeset proof for review, to ensure errors have not been introduced during production. Please review the PDF proof of your manuscript carefully, as this is the last chance to correct any scientific or type-setting errors. Please note that major changes, or those which affect the scientific understanding of the work, will likely cause delays to the publication date of your manuscript. Note: Proofs for Front Matter articles (Pearls, Reviews, Opinions, etc...) are generated on a different schedule and may not be made available as quickly.

Soon after your final files are uploaded, the early version of your manuscript, if you opted to have an early version of your article, will be published online. The date of the early version will be your article's publication date. The final article will be published to the same URL, and all versions of the paper will be accessible to readers.

For Research Articles, you will receive an invoice from PLOS for your publication fee after your manuscript has reached the completed accept phase. If you receive an email requesting payment before acceptance or for any other service, this may be a phishing scheme. Learn how to identify phishing emails and protect your accounts at https://explore.plos.org/phishing.

Thank you again for supporting open-access publishing; we are looking forward to publishing your work in PLOS Pathogens.

Best regards,

Sumita Bhaduri-McIntosh

Editor-in-Chief

PLOS Pathogens

orcid.org/0000-0003-2946-9497

Michael Malim

Editor-in-Chief

PLOS Pathogens

orcid.org/0000-0002-7699-2064
